# Giving credit to reforestation for water quality benefits

**DOI:** 10.1371/journal.pone.0217756

**Published:** 2019-06-04

**Authors:** Arturo A. Keller, Jessica Fox

**Affiliations:** 1 Bren School of Environmental Science & Management, University of California Santa Barbara, Santa Barbara, CA, United States of America; 2 Electric Power Research Institute, Palo Alto, CA, United States of America; Imperial College London, UNITED KINGDOM

## Abstract

While there is a general belief that reforesting marginal, often unprofitable, croplands can result in water quality benefits, to date there have been very few studies that have attempted to quantify the magnitude of the reductions in nutrient (N and P) and sediment export. In order to determine the magnitude of a credit for water quality trading, there is a need to develop quantitative approaches to estimate the benefits from forest planting in terms of load reductions. Here we first evaluate the availability of marginal croplands (i.e. those with low infiltration capacity and high slopes) within a large section of the Ohio River Basin (ORB) to assess the magnitude of the land that could be reforested. Next, we employ the Nutrient Tracking Tool (NTT) to study the reduction in N, P and sediment losses from converting corn or corn/soy rotations to forested lands, first in a case study and then for a large region within the ORB. We find that after reforestation, N losses can decrease by 40 to 80 kg/ha-yr (95–97% reduction), while P losses decrease by 1 to 4 kg/ha-yr (96–99% reduction). There is a significant influence of local conditions (soils, previous crop management practices, meteorology), which can be considered with NTT and must be taken into consideration for specific projects. There is also considerable interannual and monthly variability, which highlights the need to take the longer view into account in nutrient credit considerations for water quality trading, as well as in monitoring programs. Overall, there is the potential for avoiding 60 million kg N and 2 million kg P from reaching the streams and rivers of the northern ORB as a result of conversion of marginal farmland to tree planting, which is on the order of 12% decrease for TN and 5% for TP, for the entire basin. Accounting for attenuation, this represents a significant fraction of the goal of the USEPA Gulf of Mexico Hypoxia Task Force to reduce TN and TP reaching the dead zone in the Gulf of Mexico, the second largest dead zone in the world. More broadly, the potential for targeted forest planting to reduce nutrient loading demonstrated in this study suggests further consideration of this approach for managing water quality in waterways throughout the world. The study was conducted using computational models and there is a need to evaluate the results with empirical observations.

## Introduction

Reforestation and afforestation of crop and pasture fields can result in a number of benefits, including carbon sequestration [1–5]; reduced greenhouse gas emissions; reduced export of nitrogen [6], phosphorus, sediments [7–12] and sediment-associated pesticides; reduced in-stream temperatures; and increased habitat for a wide range of species [13]. Even the incorporation of buffer strips of trees between a waterbody and crop or pasture fields can yield many benefits. However, to date the evaluation of the water quality benefits from reforestation has been mostly qualitative, making it challenging to establish programs that generate water quality credits for reforestation and afforestation. The credit must take into consideration the temporal aspects associated with both reforestation per se and the water quality benefits, in addition to the characteristics of the site (e.g. soils, slope, previous landuse, etc.).

The motivation for converting croplands to forest may be to generate carbon and water quality credits, support broader biodiversity, or perhaps it is driven by the desire to produce forestry products in marginal cropland; farmers need to evaluate the value of the credits or forestry products vs. the value of the crops that can be grown on the particular property [3,4,14–19]. The economic incentive for a water quality trading program comes from funds provided by credit buyers, who have specific incentives to reduce nutrient and sediment loads in a particular region [20–23]; the program needs to be properly designed to create appropriate incentives [24]. In a water quality trading program, a credit is generated by an action that reduces the load, for example in total nitrogen (TN), by a given amount (e.g. 100 kg/yr). Credits can be generated by the implementation of Best Management Practices (BMPs), or in the case of reforestation by a land use change from cropland to forest. A well-managed water quality trading program has means for estimating credits, considering in-stream attenuation factors [25], verification of the BMP installation, and ongoing validation of credit legitimacy.

A number of water quality trading programs are in the process of being implemented or have been developed around the U.S., for example in the Ohio River Basin Water Quality Trading Project [25,26], the Chesapeake Bay [27–29], Minnesota [30,31] and in Oregon [27,28]. For a number of agricultural BMPs such as cover crops, reduced tilling, fertilizer timing and rate, tile drainage and others, practitioners use the STEPL [32] or the USEPA Region 5 [33] models to generate credit estimates. However, the conceptual model and processes considered in those models are fairly simplified and do not take into account complexities associated with a growing forest that would allow for appropriate consideration of sediment and nutrient processes. In addition, those models don’t represent forest regrowth and its implications for sediment and nutrient processes. Thus, there is a need to consider a better approach for estimating the water quality credits that reforestation projects may provide.

Rather than creating a new model, we opted for using an existing model that could consider both the dynamics of forest regrowth on a previously cultivated field and the implications for water quality in terms of nutrient load reductions. We considered a number of forest biogeochemistry models such as Forest-DNDC [34] and the related PnET [35] and DNDC [36,37] models; FORECAST [38]; BIOME-BGC [39]; MC1 [40] with the related MAPSS [41] and CENTURY [42,43] models. WARMF is a watershed scale biogeochemical model, not suitable for estimating land use changes at the field plot scale [44,45]. We have used WARMF to model the larger scale impacts of implementing BMPs on water quality, when BMPs are implemented at a much larger scale [46,47]. In addition, we considered watershed water quality models such as the Soil-Water Assessment Tool (SWAT) [48–51], which builds on several models developed over more than 3 decades at the USDA’s Agricultural Research Service [52] including EPIC (originally Erosion Productivity Impact Calculator, renamed Environmental Policy Integrated Climate) [53], a crop growth submodel, which can be parameterized for many crops, including several tree species); and APEX [54], which contains many of the submodels present in SWAT, including EPIC and ALMANAC [55,56], a more detailed crop growth model. However, most of these models, with the exception of SWAT and APEX, do not model both N and P, and thus were not considered further. In order to facilitate the use of APEX and spatial USDA soil property databases, a web-based graphical user interface was developed, denominated the Nutrient Trading Tool [57–60], and later enhanced and renamed the Nutrient Tracking Tool (NTT) [61]. Since this interface greatly facilitates access, not only to modelers but also farm owners and others involved in credit estimation, we opted for using NTT/APEX as the simulation tool for this work.

NTT has been employed to evaluate a number of conservation practices [62]. In a specific case study, the model was able to estimate the monthly and annual water yield within 15% [62]. To address questions with regards to parameterization and validation of the APEX model embedded in NTT, a framework was developed by the USDA [63,64].

APEX was designed to model the interaction of crops with hydrologic and nutrient processes, explicitly including N and P. APEX routes water, sediment, nutrient, and pesticides from their entry to the farm plot, to their exit to atmosphere, receiving waters or deep soil, depending on their processes. Hydrology is simulated using the Curve Number approach. The complete nitrogen (N) cycle is simulated in APEX, including atmospheric N inputs; fertilizer and manure N applications; crop N uptake; denitrification; mineralization; immobilization; nitrification; ammonia volatilization; organic N transport on sediment; and nitrate-nitrogen losses in leaching, surface runoff, lateral subsurface flow, and tile flow [65]. Soluble P in runoff is a linear function of soluble P loss in the top soil layer, runoff volume, and a linear adsorption isotherm. Sediment transport of P is simulated with a loading function that considers sediment yield, organic P loss in the top soil layer, and a P enrichment ratio. The model contains a database of more than 100 crops including vegetables, grasses and tree species. APEX can simulate a number of management practices, including irrigation, drainage, furrow diking, buffer strips, terraces, waterways, fertilization, manure management, lagoons, reservoirs, crop rotation and selection, pesticide application, grazing, and tillage. APEX can also be applied to evaluate land management strategies such as filter strip impacts on pollutant losses from upslope crop fields, intensive rotational grazing scenarios, vegetated grassed waterways in combination with filter strip impacts, and land application of manure removal from livestock feedlots or waste storage ponds [66]. More than a dozen agricultural BMPs have been simulated (pre- and post-implementation) using the APEX model, demonstrating the usefulness of the model for TN and TP water quality credit assessment [67].

The original APEX was modified to explicitly consider forest management [68] by including or improving rainfall interception by canopy, surface litter water balance, quick return flow, soluble N and P movement in the subsurface, and different N and P enrichment ratios. Parameters related to forest growth and nutrient utilization were calibrated and then evaluated against observed data from nine forested catchments (each with 2.6 to 2.7 ha) in eastern Texas planted with short−leaf pine and a pine−hardwood tree mixture [68]. Three catchments were undisturbed controls, and the other six represented 2 types of management practices (clearcutting followed by shearing, windrowing, and burning and clearcutting followed by roller chopping and burning). APEX simulated 37 years, from pine tree planting to the end of the application of the management practices. The last 5 years of simulation results were compared against monthly observed water quality data. Simulated average annual storm runoff ranged from -16% to 30% of observed, with a Nash-Sutcliffe Efficiency > 0.74 in all nine catchments. Annualized sediment transport ranged from -22% to 13%. While simulated nitrate and organic N export differed substantially from observed data for both clearcutting practices, for the controls the modified APEX was within 14% to 32% and -3% to 8% respectively. When nitrate and organic N exports are summed, the difference between simulated and observed decreases to 0% to 13% for the control plots, and in general decreases for the managed plots. Total P export ranges from -25% to -2% for the control plots, although the model consistently over-predicted soluble P (36% to 61%) while under-predicting bound P (-34% to -2%). A significant challenge for any model is the sparseness of monitoring data to adequately adjust parameter values in a systematic manner. Given this limitation, the APEX model performs reasonably well for these forest plots.

APEX has also been used to simulate the effectiveness of forested vegetation buffers and compared to monitored physical systems [69,70]. Four fields (0.5 to 0.9 ha) planted with grasses and actively managed for grazing were evaluated, two with no buffer and two with the forested buffer. While nutrient removal was not evaluated, a 15 m wide buffer reduced runoff on average around 36%, which would reduce dissolved N and P losses by at least that much. Sediment transport through the forested buffer was reduced by 49%, which would reduce the transport of adsorbed P in a corresponding amount. These results are clearly a function of soil properties, slope, and land use, but it indicates the significant potential to reduce nutrient transport, and the need to develop quantitative relationships to estimate the reductions. An evaluation of the sustainable forestry initiative (SFI) practices was performed using APEX [69,70]. Maintaining an unharvested hardwood plantation resulted in the lowest runoff and sediment yield, compared to pine tree clear-cutting or thinning (selection), highlighting the value of SFI practices in forests to reduce water quality impacts.

While undoubtedly there have been many reforestation projects around the world, there are very few published studies indicating the results of monitoring of nutrient export reductions, particularly over the long term. In addition to the studies mentioned before to evaluate APEX, there have been studies in Australia [71,72], China [73,74], Lower Mississippi [75], and Germany [76]. Overall, nutrient load reductions following reforestation have been substantial, on the order of 20 to 85%, depending on conditions, soil type and other factors.

In this study we first evaluated the availability of farm lands with low infiltration capacity and high slopes, as defined in the methods section, for the northern watersheds of the Ohio River Basin (Figures A and B in S1 File). These farm lands are typically less suitable for crops, and are thus the best targets for considering reforestation. We also identified those farms which could benefit even from adding a riparian forested buffer strip between the farm and an adjacent waterway. We then used the NTT/APEX model to evaluate the water quality benefits of converting croplands to forest for farms in this region.

## Methods

### Prioritizing areas for reforestation

In order to guide the selection of fields that would result in the highest water quality credits if converted from cropland to forested areas, an analysis of the current land use, soils and slopes was performed for the northern watersheds of the Ohio River Basin. For current landuse, the USDA Crop Data 2014 data layer [77] was used. Hydrologic group for each soil was obtained from the USDA SSURGO soils database[78]. The hydrologic group indicates the ability to infiltrate water, with A being soils with high infiltration capacity and thus low runoff potential, B being soils with moderate infiltration capacity, and C and D being soils with low infiltration capacity [79]. Slopes were determined from the USGS DEM model[80]. Fields with a high average slope and low infiltration capacity (e.g. clayey soils) are more likely to result in runoff of nutrients [73,76], and therefore are targets for reforestation. In many cases, clayey soils are not optimal for cropland as well, particularly in this region that receives substantial precipitation [81,82]. Table 1 presents the scores given to a field site based on slope (%) and hydrologic group. Fields with the lowest overall scores were considered as high priority.

**Table 1:** Scores used to define reforestation priorities, based on slope and hydrologic soil group.

Slope	%	Score	Infiltration	Hydrologic Soil Group	Score
Highest	>7	1	High	A	3
High	4–7	2	Medium	B, B/D, A/D	2
Medium	2–4	3	Low	D, C/D, C	1
Low	<2	4			

Using these criteria (i.e. slope, hydrologic group and current landuse), farmland in a wide area within the Ohio River Basin was analyzed to determine the potential for reforestation, either for entire fields, or for a 10 m forested buffer strip along the edge of a particular field that borders a stream or drainage ditch, whether it is used for cropland or is a grassy area. Only farmland currently used for corn or corn/soybean rotation was considered, given that a preliminary analysis indicated a lower benefit from converting pastures to forest. The objective was to identify areas of highest priority for reforestation, obtaining the maximum water quality benefit (i.e. more credits) for a similar investment in reforestation.

### Determining the magnitude of water quality credits

The NTT/APEX model was used to develop a case study of the potential water quality benefits for a hypothetical farm site in the Ohio River Basin, and then the model was applied to more broadly evaluate the range of benefits that could be achieved throughout a large region of the basin, considering 1,653 soil types and weather conditions representative of the northern Ohio River Basin, as depicted in Figure A in S1 File.

For the case study, a site was selected within the Upper Ohio River watershed, a region defined according to the USGS Hydrologic Unit Code (HUC) system as a HUC4, to better understand the benefits from implementing a reforestation scenario, when the prior landuse was corn or a corn/soybean rotation. No additional land use changes were considered. The site was selected as an example of a highest priority location, with low infiltration soils (hydrologic group C) and high slope (7%). The case study served to understand the temporal aspects related to the water quality benefits, including seasonality and interannual variability.

For a farm-specific simulation, the first step is to locate the field of interest using the Graphical User Interface (GUI) provided by NTT. The user can navigate via the map-based interface or the specific address to the location of interest. A field that had been identified as “highest priority”, based on the slope and hydrologic soil group, was selected as an example. Next, the GUI uses the USDA SSURGO soils database to obtain the soil property values for the area of interest. Table 2 presents the values for the case study farm site. The next step is to select a crop type. NTT allows the user to select from a wide range of crops, including corn or corn/soybean rotation, as well as different tree species. For the case study, a pine tree plantation was considered, and compared against corn or a corn/soybean rotation. Typical fertilizer application was considered for the corn and corn/soybean rotation, but no fertilizer was added to the reforested land (see Tables C, D, and E in S1 File for more details on agricultural operations). A full conversion of the crop field to reforested land was assumed for this analysis. Crop properties are selected from the database, including nutrient requirements, growth rates and yields. For each crop type, a management file was created to consider the typical timing of various crop operations such as tillage planting, irrigation, fertilization and harvesting. For the pine plantations no harvesting was considered. NTT also uses a weather generating algorithm, based on the weather parameters for the weather station nearest to the site. The underlying weather data is from PRISM [85] and includes minimum and maximum air temperature, mean wind speed, mean net radiation, and precipitation. Each NTT simulation considers a 35-year period, which typically captures the variability in weather from dry to wet years. This also allows for a “warm-up” period, and in the case of the reforestation scenario, allows the trees to grow to maturity. The last 12 simulated years are presented in the results.

**Table 2:** Soil properties for the case study (from websoilsurvey.sc.egov.usda.gov).

Soil	Composition	Texture	Soil Organic Matter	Hydraulic Conductivity (mm/hr)	Bulk density (g/cm^3^)	Slope
Upshur-Gilpin	69.87%	Silty clay	2.25%	8.80	1.35	6.5–6.9%
Vandalia-Gilpin	30.13%	Silty clay loam	2.00%	2.65	1.32	7.8%

For the evaluation of a large number of sites (1,653 combinations of soils and weather conditions), a script was used to run the APEX model with soils selected at random throughout the northern Ohio River Basin. While soils and weather conditions vary substantially through the entire basin, this provides an idea of the range of water quality benefits that may be available. Full conversion from cropland to forested land was assumed. The results were compiled and the summary statistics reported herein.

## Results

### Prioritizing reforestation

**Fig 1:**
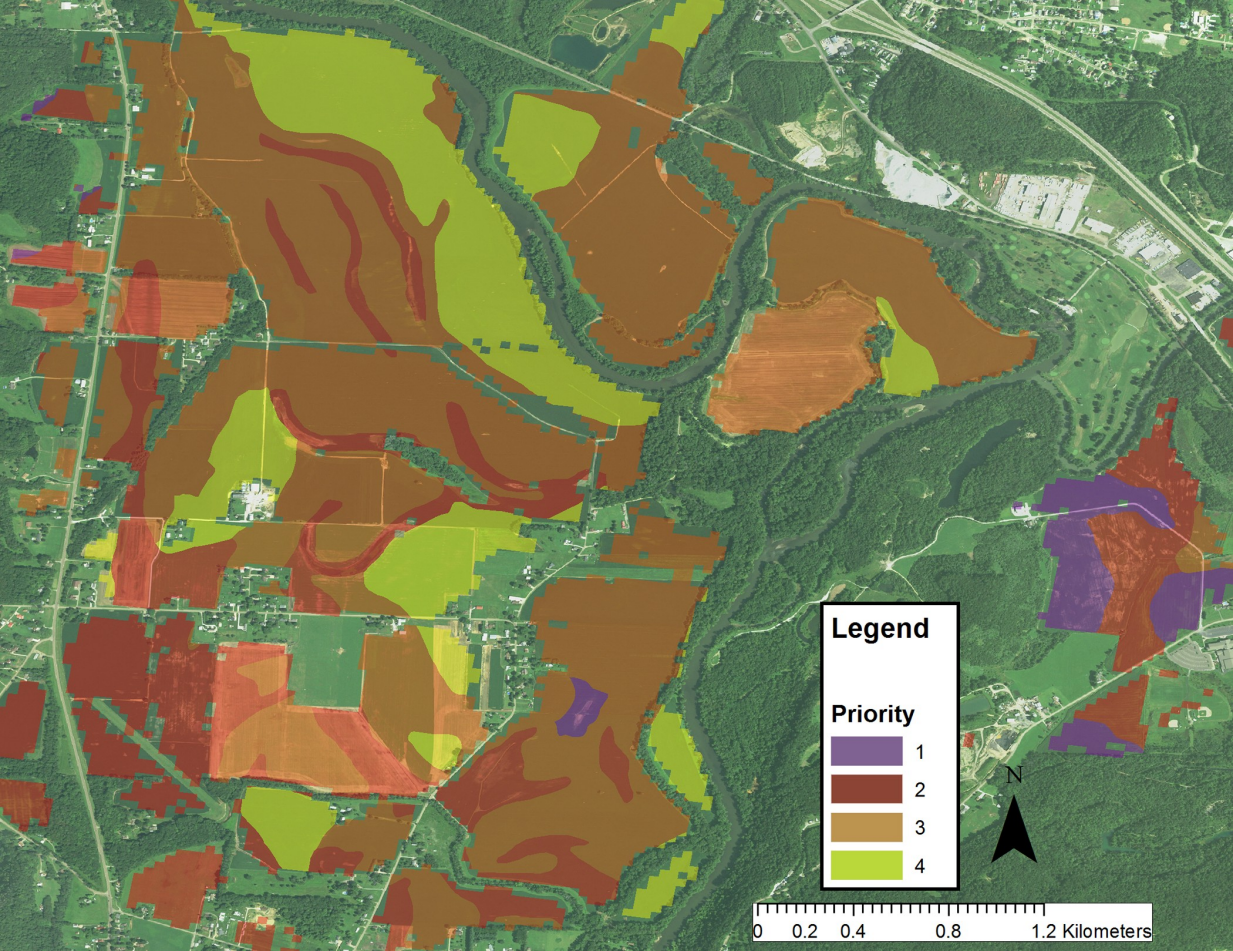
Priority analysis of cropland fields in a region of the Scioto River watershed. Color code is: highest = purple, high = red, medium = orange/brown, and low priority = yellow green.

**Fig 2:**
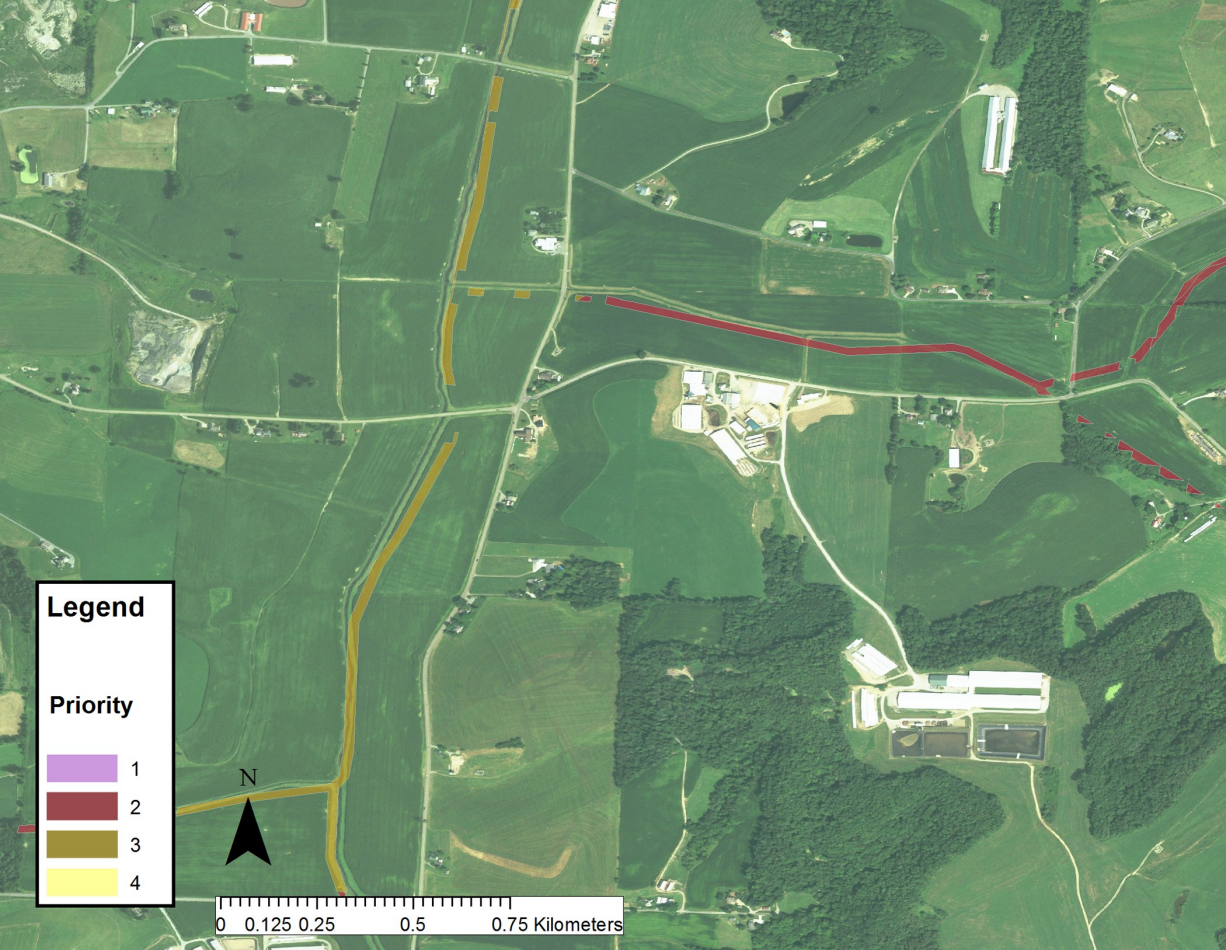
Priority analysis of buffer strips for croplands in a region of the Scioto River watershed. Color code is: highest = purple, high = red, medium = orange/brown, and low priority = yellow.

An example in the Scioto River watershed, another large HUC 4 watershed within the Ohio River Basin, of the evaluation of cropland that would be high priority for reforestation is shown in Fig 1. In this image, urban and currently forested areas are not considered. Cropland was prioritized and color coded. Fields with combined scores < 3 were assigned highest priority (purple), score of 3 have high priority (red), score of 4 are medium (orange) and 5 and greater are low priority (yellow). In this example, the majority of the fields fall within low and medium priority, but there are a significant number of fields in the high and highest priorities, particularly near waterways. In some cases, the farmland owner may be only interested in implementing a forested buffer strip; an example in the same watershed is shown in Fig 2, indicating the potential locations for buffer strips and their priority based on this analysis. A detailed analysis of several watersheds in the northern part of the Ohio River Basin (Table 3) indicates that approximately 32% of the total land is covered by cropland, and a large fraction (61%) of the cropland is in low infiltration soils. Combining the infiltration and slope data indicates that approximately 14% of the farmland in this watershed is of high or highest priority for reforestation and an additional 35% would be a mid-priority, for a total of 49%. If all the middle, high and highest priority forested buffer strips were implemented, they would occupy only 0.4% of the total cropland in this region, with a minor effect on crop production. The 0.4% reflects the fact that many croplands in this region already have partial vegetated buffer strips. In some watersheds with a greater fraction of cropland (e.g. Scioto River watershed) and low infiltration soils in cropland areas, the proportion of croplands amenable for reforestation (highest priority) is 9.4%. These are usually marginal lands with very clayey soils and high slopes.

**Table 3:** Availability of high priority cropland fields for reforestation in the northern Ohio River Basin.

Ohio River Basin (north of Ohio River)			
	Area	Area	
	(km^2^)	(1,000 acres)	(%)
Total Land	225,669	55,764	100%
Cropland	71,765	17,733	32%
Forests/Wetlands	91,572	22,628	41%
Urban	23,945	5,917	11%
Rangeland	30,568	7,554	14%
Other	7,819	1,932	3%
Cropland Infiltration			of total cropland
High (Hydrologic Grp A)	1,652	408	2%
Medium (Hydrologic Grp B, B/D, A/D)	25,476	6,295	35%
Low (Hydrologic Grp D, C/D, C)	44,101	10,898	61%
Missing Data*	536	133	1%
Cropland Slope			of total cropland
Low (<2%)	47,877	11,831	67%
Medium (2–4%)	10,321	2,550	14%
High (4–7%)	8,992	2,222	13%
Highest (>7%)	4,575	1,131	6%
Crop Land Priority For Conversion			of total cropland
Highest Priority Funding	6,007	1,484	8%
High Priority Funding	4,140	1,023	6%
Mid Priority Funding	25,127	6,209	35%
Low Priority Funding	35,955	8,885	50%
Missing Data*	536	133	1%
Crop Land Buffer Zone Priority For Conversion			of total cropland
Highest Priority Funding	46.0	11.4	0.06%
High Priority Funding	34.3	8.5	0.05%
Mid Priority Funding	208.4	51.5	0.29%
Low Priority Funding	298.2	73.7	0.42%
Missing Data*	8.3	2.1	0.01%

*missing data: data on soil infiltration capacity not available

### Determining the magnitude of water quality credits

#### Farm-level reduction in fluxes from reforestation

**Fig 3:**
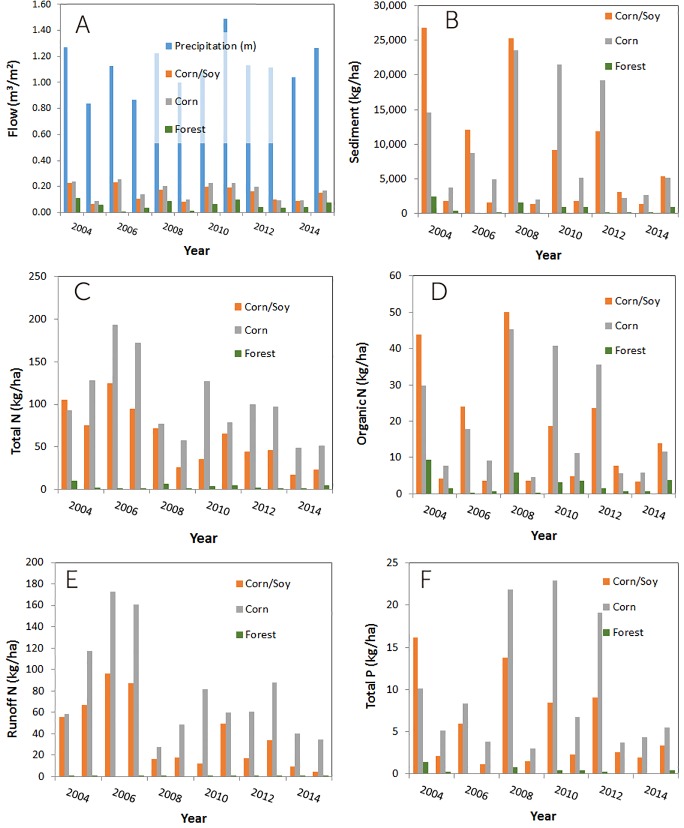
Annual trends in (A) water, (B) sediment and (C-F) nutrient fluxes at a farm in the Upper Ohio River HUC4, with soil in hydrologic group C and 7% average slope, for three different land uses (corn/soybean rotation, corn only, and forested).

At the farm site-specific case study in the Upper Ohio River HUC4, with moderate infiltration soils and high slopes, there are significant annual differences in runoff, sediment and nutrient fluxes out based on land use (Fig 3). The modeled results and statistics are presented in Tables A (annual) and B (monthly average) in S1 File. Precipitation, normalized by area, ranges from slightly over 0.8 m/yr to almost 1.5 m/yr over the last twelve years of simulation using NTT. The APEX model within NTT predicts that about 0.1 to 0.26 m/yr of runoff is generated annually if the farm is planted with corn or a corn/soybean rotation, but decreases to 0.002 to 0.11 m/yr if forested. There is an average decrease in runoff of 64–68% by converting from these crops to forested areas. There is no direct correlation between annual precipitation and annual runoff, since there a several factors that control runoff, including antecedent moisture conditions before storms, evapotranspiration, soil type and infiltration, and vegetative soil cover. Sediment export is predicted to vary substantially year to year, from a minimum of 1,345 kg/ha-yr to a maximum of 25,300 kg/ha-yr, in part due to the variability in runoff, and also the state of soil cover during the major storms that result in substantial erosion. Nevertheless, there is a marked decrease of 92–93% in mean annual sediment export if the field is reforested, which is the main driver for the reduction in mean annual export of TP (96–97%) and organic N (84–86%). N in runoff (mostly as nitrate) is minimal from forested lands, averaging 0.3 kg N/ha-yr, compared to 38–79 kg N/ha-yr from croplands. Thus, the reduction in TN is a combination of the reduced export in runoff and the decrease in organic N, for a decrease in export of 95–97% on average. There is considerable interannual variability in the reduction in TN export, from 48 to 193 kg N/ha, which is important to consider in credit calculations and monitoring. Annual TN export from a corn plantation in this field is 102 ± 46 kg/yr, but only 3.1 ± 2.9 kg/yr once the field is reforested. While not as large in absolute magnitude, there is also noticeable annual variability in TP reduction, from 1 to 15 kg P/ha. Nutrient reduction validation may require monitoring over a long enough period of time to capture the variability. While these results are specific to this location, they demonstrate the significant effect of converting marginal croplands, with low infiltration soils and high slopes to forested areas, and the need to consider the interannual variability in the credit calculation.

**Fig 4:**
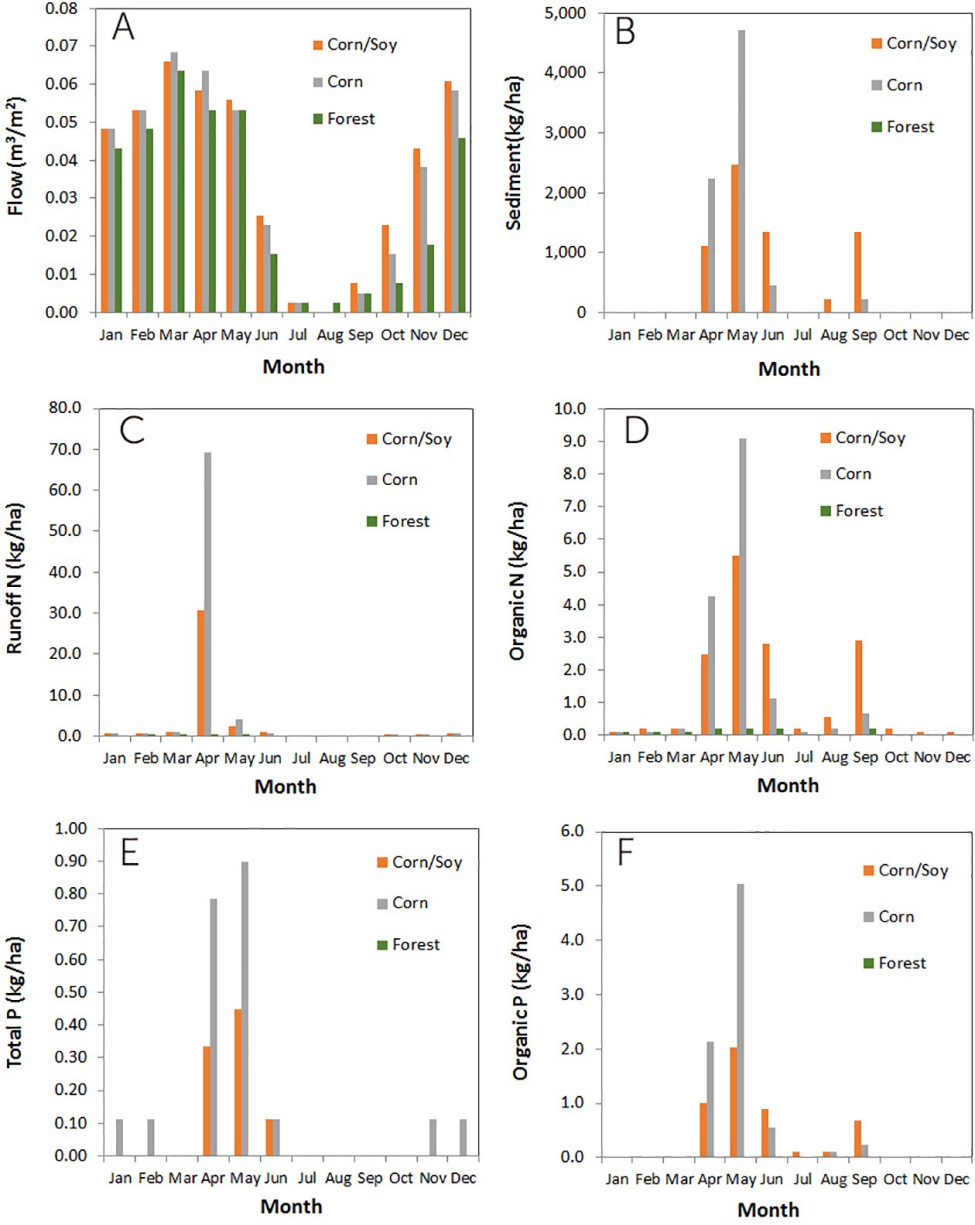
Monthly trends in (A) water, (B) sediment and (C-F) nutrient fluxes at a farm in the Upper Ohio River HUC4, with soil in hydrologic Group C and 7% average slope, for three different land uses (corn/soybean rotation, corn only, and forested).

This case study also serves to understand the monthly variability in fluxes (Fig 4). These are the average monthly fluxes of the 12 years presented in Fig 3. On average, runoff flow crests in March due to snowmelt, and is minimal in August. However, the peak runoff month varies year-to-year, smoothing out the fluxes. Sediment transport is highest in April and May for corn and corn/soybean rotations, and is on average much smaller for forested lands. Organic N and P are strongly driven by sediment transport, while runoff N is highest during April, lagging the peak flow by one month, based on the application of fertilizer during planting. As expected, the large peaks in nutrient fluxes observed for the croplands almost disappear when the same field is forested. The timing of the fluxes and corresponding flux reductions needs to be taken into account in the development of a water quality trading program, as well as monitoring of the benefits of implementing load reductions.

### Large scale reduction in fluxes from reforestation of marginal croplands

**Fig 5:**
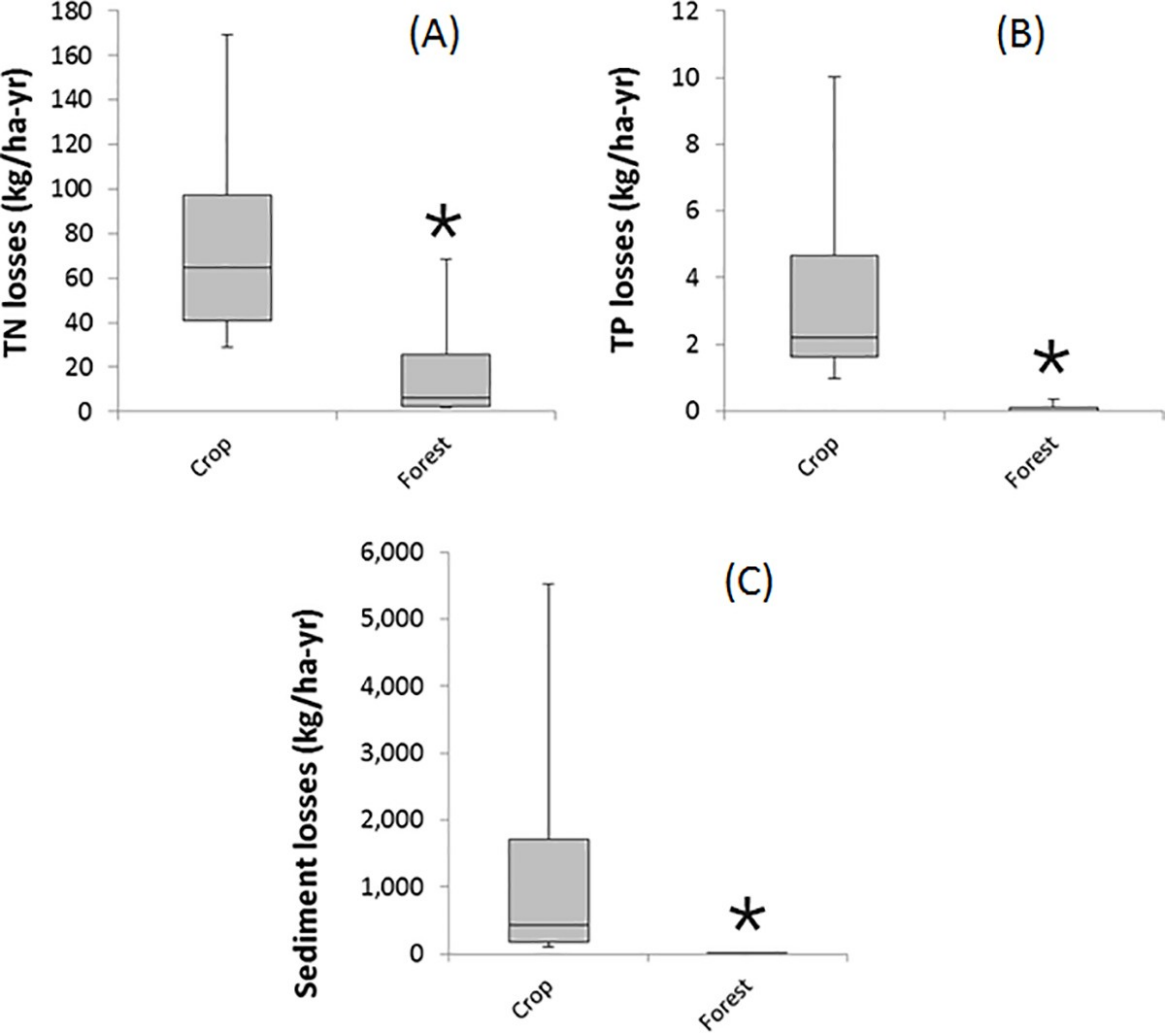
Export of (A) TN, (B) TP, (C) sediments from corn fields (crop) compared to the same field used for forest, for n = 1,653 fields in the northern Ohio River Basin. Differences are statistically significant (p < 0.01).

In order to determine the variability due to soil types and meteorological conditions, a similar comparison between crop lands (corn/soybean rotations) and reforested fields was conducted for 1653 representative areas in the northern region of the Ohio River Basin (Fig 5 and Table F in S1 File). The median reduction in TN export due to reforestation is 90%, from 64.6 to 6.3 kg N/ha-yr. However, there is considerable variability in TN losses, given that even sites with similar soil types may have very different average slope and meteorological conditions. For TP, the median reduction is even higher, greater than 99%, due to the minimization of sediment export, the main pathway for P export. For the three water quality indicators (TN, TP, sediments), the differences are statistically significant (p < 0.01). If the median load reductions were to be achieved in the high and highest priority fields (Table 2), it could result in an annual decrease of 60 million kg N and 2 million kg P reaching the streams and rivers of the northern Ohio River Basin, which eventually drains to the Gulf of Mexico.

## Discussion

We demonstrated the value at the individual farm level using a case study, which also served to highlight the need to take a longer perspective in terms of the calculation of the water quality credits. Ideally, long term in-stream monitoring to verify modeling results and therefore credit calculations can also be done. The results indicate that there are important interannual and monthly variations in nutrient fluxes, particularly when the land is used for crops. Reforestation influences the fluxes in various ways. First, there is considerable retention of the precipitation that passes through the canopy, such that surface runoff decreases considerably, as shown in Fig 3A, decreasing water flux out. This in turn reduces soil erosion, which decreases sediment fluxes out of the reforested field, thereby reducing release of sediment-attached phosphorous (Fig 3B). Overall, by reducing the transport pathways, both TN and TP fluxes out of the field decrease considerably (Fig 3C and 3F). Once the land is forested, the variations are actually minimal since all fluxes are very small. Given these fluctuations in nutrient fluxes, a trading program would have to determine the expected annual average decrease in nutrient fluxes, with a margin of safety [25]. Further, there would be an expectation that the reforested land would be kept forested for a sufficiently long period (e.g. current landowner contracts in the EPRI Ohio River Basin Water Quality Trading Project range from 20 to 40 years for forest planting).

The spatial variability was evaluated by performing a large-scale analysis of more than 1,650 sites in Ohio and Indiana. Given the large variation in soils and meteorological conditions across this large area, there is a significant range in nutrient reductions from conversion to corn/soy rotation to forest, with the 25^th^ and 75^th^ percentile ranging from 39 to 72 kg N/ha-yr and 1.6 to 4.5 kg P/ha-yr. Given the wide range in results, it will be useful to critically evaluate the actual reductions at a number of farm sites, and using a variety of methods and models, to build confidence in the modeled reductions.

The results from this study compare well to other studies. For example, a study in the Lower Yazoo River Watershed, in Mississippi, BASINS-HSPF predicted that reforestation of agricultural lands bordering or near streams would substantially reduce nitrate and phosphate loads [9], by up to 60 and 4 kg/ha-y, respectively. These estimates are very similar to those predicted by NTT. While BASINS-HSPF is a good simulation tool for watershed and catchment scale studies, it would be impractical for modeling the credits from individual farms, given the significant level of effort (several days) to setup the model. In a study in the Swan and Canning Rivers, Western Australia, full reforestation of agricultural land was estimated to reduce phosphorus and nitrogen export by 50 and 85% [71], respectively, which is of similar magnitude as estimated in our study considering a broader area and not just an individual farm. Similarly, a study in Australia [72] found that the difference between a fully forested river channel network and one with no vegetation cover would be an increase (from reforested to no cover) of 50 and 200 times in sediment yield per unit area, 25 and 60 times for TP; and 1.6 and 4.1 times for TN; this highlights the role of reforestation with regards to retaining sediments and nutrients, as simulated by NTT. A modeling study in the Elbe River, Germany, found that reforestation of croplands could result in TP reductions of up to 60% in some catchments; this did not consider all croplands in a given catchment, only those deemed less suitable based on slope and soil properties [76]. In support of the NTT results with regards to sediment export and the associated nutrients, a study of reforestation projects in southeastern China estimated dramatically reduced annual total soil loss, from 53 to 256 tons/ha-yr before reforestation to 2–43 tons/ha-yr after reforestation [73]. In another study in the Three Gorges region, China, reforestation of sloped cropland decreased the TN load by 69% and the TP load by 82% [74].

The Ohio River Basin has many soils that have low infiltration capacity and given the relatively high precipitation, the response to reforestation is substantial. Other regions with highly permeable soils, or much lower precipitation, or where reforestation is not supported by the local conditions, may not observe the same level of flux reductions. While there is a need to verify the local conditions, NTT can be easily applied to study the water quality benefits of reforestation for each specific field, not only in the Ohio River Basin, but wherever there are available datasets that support NTT.

## Conclusions

Using easily accessible information from U.S. agencies (USGS and USDA), we conducted an analysis of hundreds of thousands of farms in the northern region of the Ohio River Basin, to determine which fields have the highest potential for reducing TN and TP release from reforestation, based on their current landuse, slope and hydrologic soil group. This general analysis approach could be extended to farms internationally, to identify priority areas for reforestation in terms of water quality benefits. Naturally, reforestation would provide many other benefits (e.g. carbon sequestration, biodiversity, forest products). Even just in the study region, up to 14% of the current croplands (more than 10,000 km^2^) are in these marginally productive locations, representing a significant potential for conservation with minor impact on crop production. These areas also are the most significant contributors to runoff, sediment and nutrient transport, given their characteristics. Therefore, there is a compelling reason to consider their reforestation at a large scale.

If one could achieve the conversion of all the high and highest priority lands, the potential reduction in nutrient loads to the Ohio River, its tributaries and smaller streams would be on the order of 60 million kg N/yr and 2.2 million kg P/yr. A recent study estimated that the loads in the Ohio River (at its discharge to the Mississippi River in Metropolis, IL) are approximately 384–650 million kg N/yr and 36–63 million kg P/yr [83], which means that these reductions would be on the order of 12% for TN and 5% for TP, for the entire basin. This could have a noticeable and lasting beneficial impact on these receiving water bodies, all the way to the Gulf of Mexico where there are long-running efforts to reduce the dead zone cause by TN and TP loading; there is currently an overall 45% TN reduction target, with an interim goal of 25% reduction by 2025 [84]. Targeting specific areas under the EPRI Ohio River Basin Water Quality Trading Project, or other nutrient management initiative, may yield significant nutrient credits and provide both local and downstream benefits. More broadly, the potential for targeted forest planting to reduce nutrient loading demonstrated in this study suggests further consideration of this approach for managing water quality in waterways throughout the world.

## Supporting information

S1 File Figures A and B, and Tables A, B, C, D, E and F.
(DOCX)
