# Supplementary material for: Giving credit to reforestation for water quality benefits
Source: PLoS One. 2019 Jun 4;14(6):e0217756. doi: 10.1371/journal.pone.0217756 (PMC6548385; doi:10.1371/journal.pone.0217756)
Supplement: S1 File — Figures A and B, and Tables A, B, C, D, E and F. (DOCX) [file pone.0217756.s001.docx]

**Supporting Information**

For

Giving Credit to Reforestation for Water Quality Benefits

By Arturo A. Keller^1^ and Jessica Fox^2^

^1^Bren School of Environmental Science & Management, University of California Santa Barbara

^2^Electric Power Research Institute, Palo Alto, CA


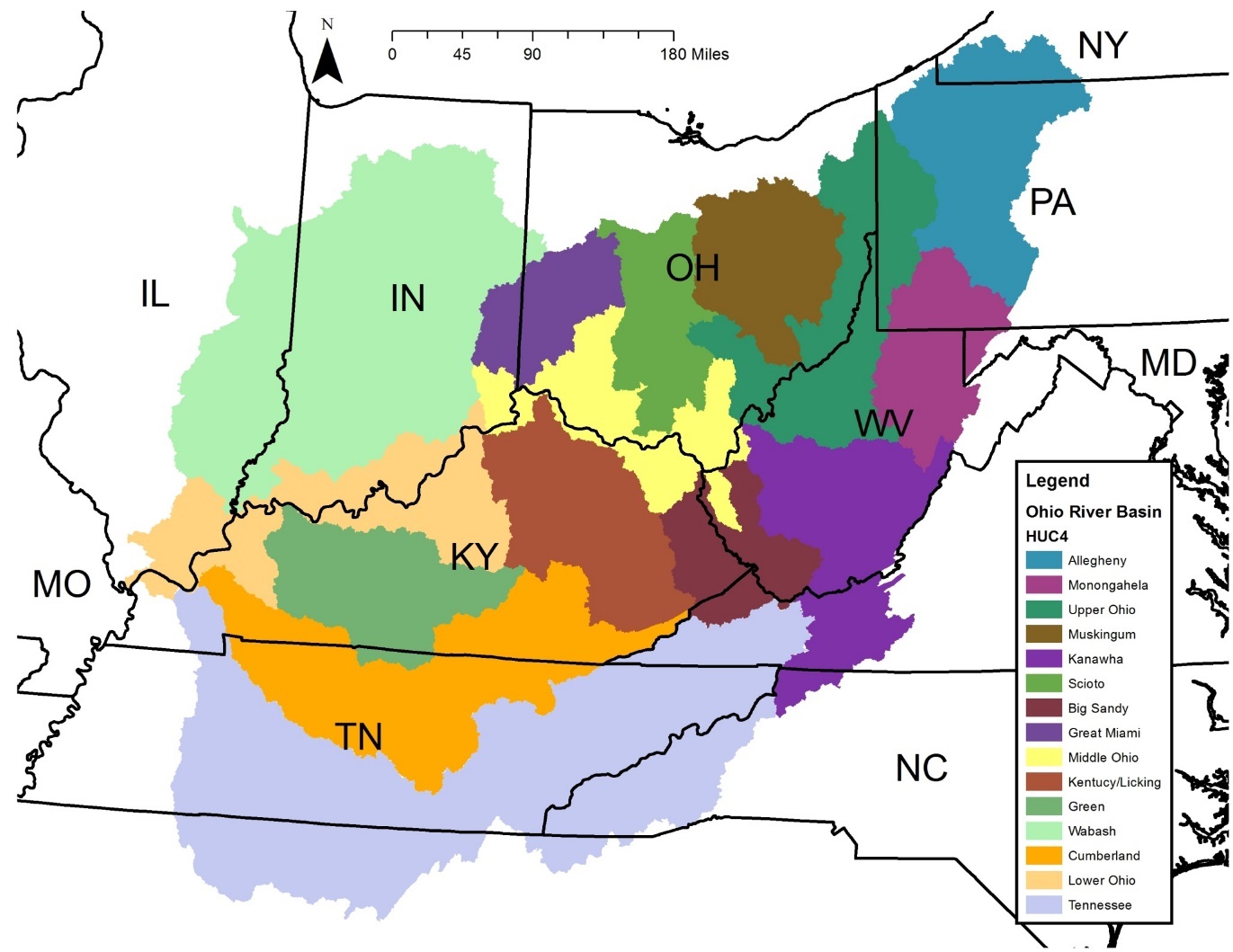


Fig A. Watersheds in the Ohio River Basin


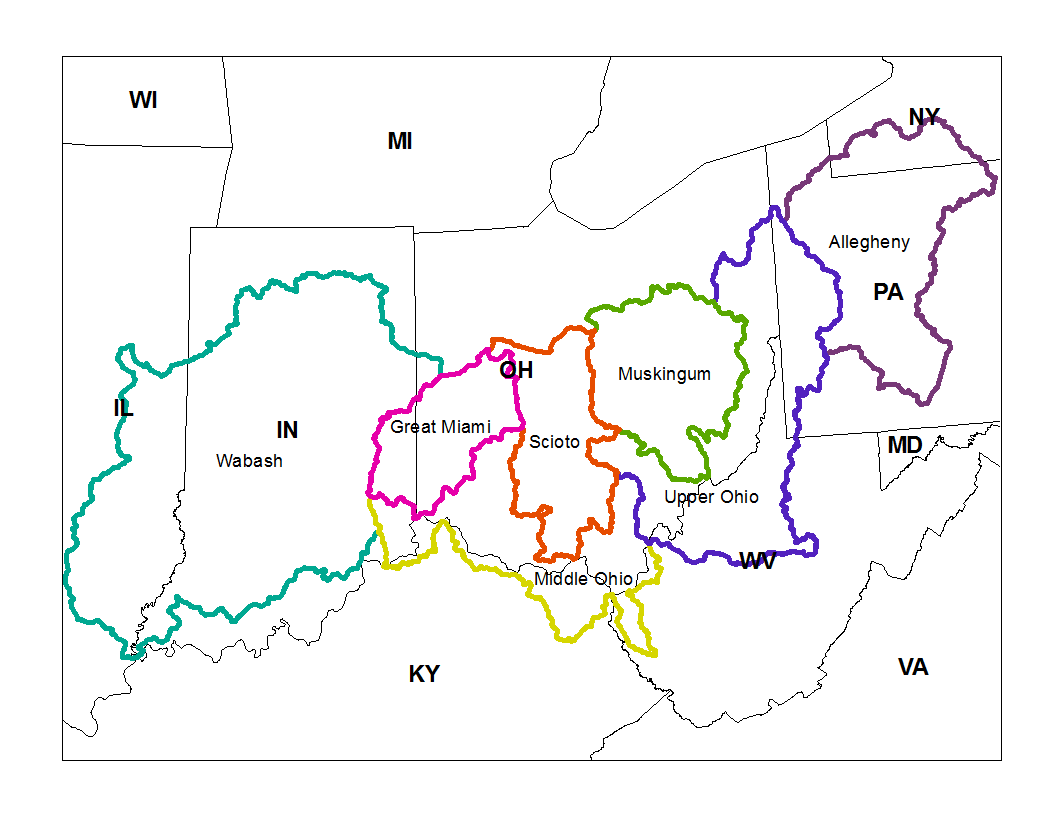


Fig B. Watersheds in the Ohio River Basin considered for the water quality trading program.

# Review of models considered for modeling water quality implications of reforestation

There are a number of models that have been explicitly designed to model forest ecosystems and their biogeochemistry. A few of them consider the effects of nutrient availability. For example Forest-DNDC [1]) was developed by USDA to quantify carbon sequestration and trace gas emissions from forest ecosystems, building on two existing models, PnET and DNDC. PnET simulates physiological processes, including forest photosynthesis, respiration, carbon allocation, and litter production [2]. PnET considers the foliar nitrogen-photosynthesis relationship explicitly [2]. DNDC was originally a soil biogeochemical model used to simulate carbon (C) and nitrogen (N) processes, such as hydraulic transport, thermal exchanges, decomposition, and denitrification that result in nitrous oxide, carbon dioxide and nitrogen gas evolution [3]. It was updated to consider crop plants and their uptake and release of C and N as a function of growth throughout the plant life-cycle [4]. The updated DNDC model also considered agricultural practices such as fertilization, irrigation, tillage, crop rotation, and manure amendments. The combination of PnET and DNDC, with some additional processes such as nitrification, soil freezing and thawing, and an active forest litter layer, results in a model that actively simulates above and below ground plant and biogeochemical processes. Forest-DNDC can predict carbon storage in soil and biomass, greenhouse gas emissions and N leaching. The model is driven by climate, soils, vegetation (type and processes) and management practices such as deforestation, reforestation, thinning, burning, drainage, wetland restoration, and fertilization. However, it does not consider phosphorus (P) processes.

The BIOME-BGC [5] model is a multi-biome generalization of FOREST-BGC [6], which is an ecosystem process model for simulating C, N and water cycling through a forest ecosystem. FOREST-BGC considers hydrological processes (e.g. canopy interception, evaporation, transpiration) as well as photosynthesis, growth and maintenance respiration, litterfall, decomposition and nitrogen mineralization. While the model has been used extensively, it does not consider transitions from an initial vegetation to a forest, and also does not consider P processes.

FORECAST is a combination of a mechanistic description of tree physiological and soil biogeochemical processes with historical bioassay data for empirical fitting, intended for forest management [7]. It considers a number of silvicultural and harvesting systems and natural disturbances (e.g. wind, pests, fire). It calculates most physiological processes at the stand level, but can disaggregates to different stand age groups. Although it does consider some N processes, such as biological fixation, effect of N on photosynthesis, and loss of nitrate via leaching and denitrification (combined without differentiation), it is limited in its ability to model the entire N cycle, and does not consider P processes.

MC1 [8] combines the physiology used in the MAPSS model [9] with a modified version of CENTURY, a biogeochemical model [10,11]. MC1 is a dynamic vegetation model, designed initially to consider the potential shifts due to climate change, but extended to consider changes to due fire and other disturbances. The biogeochemical model simulates C and N dynamics in the ecosystem with above- and below-ground processes such as hydrologic processes (e.g. interception, evaporation, transpiration, runoff, groundwater outflow, saturated flow into soil), net primary production (which depends on leaf N and other inputs), and decomposition and the release of C and N pools. As with many other models, MC1 is limited in its ability to model the entire N cycle, and does not consider P processes.

The Soil-Water Assessment Tool (SWAT) [12–15] was originally developed to model watershed-scale biogeochemical processes and the effects of agricultural practices on water quality. It builds on several models developed over more than 3 decades at the USDA’s Agricultural Research Service [16]. A key component is the EPIC (originally Erosion Productivity Impact Calculator, renamed Environmental Policy Integrated Climate) (Williams and Sharpley, 1989) crop growth submodel, which can be parameterized for many crops, including some tree species (e.g. lodgepole pine (*Pinus contorta* Douglas ex Loudon), white spruce (*Picea glauca* var. *glauca*), black spruce (*Picea mariana*), trembling aspen (*Populus tremuloides Michx.*), hybrid poplar ‘Tristis #1’ (*Populus balsamifera* L. × Populus tristis Fisch) and eastern cottonwood (*Populus deltoids* Bartr.)). Updates to SWAT in SWAT2005 consider forest growth from seedling to mature stand [13–15]. A wide range of crop management practices are considered. The model simulates not only N and P, but also sediments, pesticides, and other major ions. Biomass removal and manure deposition can account for grazing operations. A number of BMPs (conservation and management practices) can be simulated. However, SWAT requires many data sets to obtain the required parameter values, particularly for spatially and/or temporally distributed parameters (e.g. soils, landuse, meteorology, point source discharges). Thus, applying a watershed-scale model to perform a field-scale calculation requires a considerable amount of additional effort and may not be practical for calculating water quality credits for thousands (or millions) of farms in a given region.

# Simulated results for case study site

Table A. NTT/APEX output for last 12 years of simulation. All values are on a per year basis.

|  | 2004 | | 2005 | | 2006 | | 2007 | | 2008 | | 2009 | | 2010 | | 2011 | | 2012 | | 2013 | | 2014 | | 2015 | | Mean | | Standard Deviation |
| --- | --- | --- | --- | --- | --- | --- | --- | --- | --- | --- | --- | --- | --- | --- | --- | --- | --- | --- | --- | --- | --- | --- | --- | --- | --- | --- | --- |
| Precipitation (m) | | 1.273 | | 0.841 | | 1.125 | | 0.866 | | 1.227 | | 1.001 | | 1.092 | | 1.488 | | 1.133 | | 1.115 | | 1.041 | | 1.267 | | 1.12 | 0.18 |
| Runoff (m) | |  | |  | |  | |  | |  | |  | |  | |  | |  | |  | |  | |  | |  |  |
| Corn/Soy | | 0.229 | | 0.069 | | 0.234 | | 0.104 | | 0.175 | | 0.081 | | 0.201 | | 0.193 | | 0.165 | | 0.099 | | 0.086 | | 0.150 | | 0.15 | 0.059 |
| Corn | | 0.241 | | 0.086 | | 0.257 | | 0.142 | | 0.206 | | 0.099 | | 0.229 | | 0.226 | | 0.196 | | 0.094 | | 0.097 | | 0.168 | | 0.17 | 0.064 |
| Forest | | 0.112 | | 0.056 | | 0.003 | | 0.036 | | 0.084 | | 0.010 | | 0.061 | | 0.099 | | 0.041 | | 0.036 | | 0.038 | | 0.074 | | 0.05 | 0.034 |
| Total N (kg/ha) | |  | |  | |  | |  | |  | |  | |  | |  | |  | |  | |  | |  | |  |  |
| Corn/Soy | | 104.80 | | 74.76 | | 124.41 | | 94.38 | | 71.85 | | 25.78 | | 35.42 | | 65.46 | | 44.39 | | 45.84 | | 17.26 | | 23.31 | | 60.6 | 34.5 |
| Corn | | 92.36 | | 127.55 | | 193.23 | | 172.16 | | 76.33 | | 56.94 | | 126.54 | | 78.68 | | 99.87 | | 97.07 | | 48.64 | | 50.89 | | 101.7 | 46.0 |
| Forest | | 10.09 | | 1.91 | | 0.34 | | 0.90 | | 6.73 | | 0.56 | | 3.70 | | 4.37 | | 1.79 | | 1.12 | | 1.12 | | 4.26 | | 3.1 | 2.9 |
| Runoff N (kg/ha) | |  | |  | |  | |  | |  | |  | |  | |  | |  | |  | |  | |  | |  |  |
| Corn/Soy | | 55.72 | | 66.69 | | 96.28 | | 87.09 | | 16.36 | | 17.49 | | 11.88 | | 49.32 | | 16.81 | | 33.96 | | 9.64 | | 4.37 | | 38.8 | 31.7 |
| Corn | | 58.17 | | 117.35 | | 172.84 | | 160.95 | | 27.46 | | 48.76 | | 81.82 | | 60.08 | | 60.64 | | 87.65 | | 40.13 | | 34.63 | | 79.2 | 47.9 |
| Forest | | 0.67 | | 0.34 | | 0.00 | | 0.22 | | 0.45 | | 0.00 | | 0.34 | | 0.56 | | 0.22 | | 0.22 | | 0.22 | | 0.34 | | 0.30 | 0.20 |
| Total P (kg/ha) | |  | |  | |  | |  | |  | |  | |  | |  | |  | |  | |  | |  | |  |  |
| Corn/Soy | | 16.16 | | 2.12 | | 5.96 | | 1.18 | | 13.81 | | 1.49 | | 8.40 | | 2.28 | | 9.02 | | 2.59 | | 1.96 | | 3.37 | | 5.7 | 5.1 |
| Corn | | 10.12 | | 5.18 | | 8.32 | | 3.84 | | 21.89 | | 3.06 | | 22.91 | | 6.75 | | 19.07 | | 3.69 | | 4.32 | | 5.49 | | 9.6 | 7.4 |
| Forest | | 1.46 | | 0.22 | | 0.00 | | 0.11 | | 0.78 | | 0.00 | | 0.45 | | 0.45 | | 0.22 | | 0.11 | | 0.11 | | 0.45 | | 0.36 | 0.41 |
| Organic N (kg/ha) | |  | |  | |  | |  | |  | |  | |  | |  | |  | |  | |  | |  | |  |  |
| Corn/Soy | | 43.82 | | 4.15 | | 23.99 | | 3.59 | | 50.10 | | 3.59 | | 18.72 | | 4.82 | | 23.65 | | 7.73 | | 3.36 | | 14.01 | | 16.8 | 16.1 |
| Corn | | 29.93 | | 7.73 | | 17.93 | | 9.08 | | 45.28 | | 4.71 | | 40.69 | | 11.21 | | 35.53 | | 5.72 | | 5.83 | | 11.66 | | 18.8 | 14.9 |
| Forest | | 9.30 | | 1.46 | | 0.11 | | 0.67 | | 5.83 | | 0.34 | | 3.25 | | 3.59 | | 1.46 | | 0.78 | | 0.78 | | 3.70 | | 2.6 | 2.7 |
| Sediment (kg/ha) | |  | |  | |  | |  | |  | |  | |  | |  | |  | |  | |  | |  | |  |  |
| Corn/Soy | | 26,852 | | 1,793 | | 12,105 | | 1,569 | | 25,331 | | 1,345 | | 9,191 | | 1,793 | | 11,881 | | 3,138 | | 1,345 | | 5,380 | | 8,477 | 9,164 |
| Corn | | 14,571 | | 3,811 | | 8,743 | | 4,932 | | 23,538 | | 2,018 | | 21,520 | | 5,156 | | 19,279 | | 2,242 | | 2,690 | | 5,156 | | 9,471 | 8,031 |
| Forest | | 2,466 | | 448 | | - | | 224 | | 1,569 | | - | | 897 | | 897 | | 224 | | 224 | | 224 | | 897 | | 673 | 734 |

Table B. NTT/APEX average monthly output based on the last 12 years of simulation. All values are on a per month basis.

|  | Jan | Feb | Mar | Apr | | May | | Jun | | Jul | | Aug | | Sep | Oct | | Nov | | | | Dec | | Mean | Standard Deviation |
| --- | --- | --- | --- | --- | --- | --- | --- | --- | --- | --- | --- | --- | --- | --- | --- | --- | --- | --- | --- | --- | --- | --- | --- | --- |
| Surface flow (m) |  |  |  |  | |  | |  | |  | |  | |  |  | |  | | | |  | |  |  |
| Corn/Soy | 0.048 | 0.053 | 0.066 | 0.058 | | 0.056 | | 0.025 | | 0.003 | | 0.000 | | 0.008 | 0.023 | | 0.043 | | | | 0.061 | | 0.037 | 0.024 |
| Corn | 0.048 | 0.053 | 0.069 | 0.064 | | 0.053 | | 0.023 | | 0.003 | | 0.000 | | 0.005 | 0.015 | | 0.038 | | | | 0.058 | | 0.036 | 0.025 |
| Forest | 0.043 | 0.048 | 0.064 | 0.053 | | 0.053 | | 0.015 | | 0.003 | | 0.003 | | 0.005 | 0.008 | | 0.018 | | | | 0.046 | | 0.030 | 0.023 |
| Organic N (kg/ha) |  |  |  |  |  | |  | |  | |  | |  | | |  | |  |  |  | |  |  |  |
| Corn/Soy | 0.112 | 0.224 | 0.224 | 2.466 | | 5.492 | | 2.802 | | 0.224 | | 0.560 | | 2.914 | 0.224 | | 0.112 | | | | 0.112 | | 1.3 | 1.7 |
| Corn | 0.112 | 0.112 | 0.224 | 4.259 | | 9.079 | | 1.121 | | 0.112 | | 0.224 | | 0.673 | 0.000 | | 0.000 | | | | 0.000 | | 1.3 | 2.7 |
| Forest | 0.112 | 0.112 | 0.112 | 0.224 | | 0.224 | | 0.224 | | 0.000 | | 0.000 | | 0.224 | 0.000 | | 0.000 | | | | 0.000 | | 0.10 | 0.10 |
| Runoff N (kg/ha) |  |  |  |  |  | |  | |  | |  | |  | | |  | |  |  |  | |  |  |  |
| Corn/Soy | 0.560 | 0.673 | 0.897 | 30.711 | | 2.354 | | 0.897 | | 0.000 | | 0.000 | | 0.000 | 0.224 | | 0.448 | | | | 0.673 | | 3.1 | 8.7 |
| Corn | 0.560 | 0.673 | 0.897 | 69.381 | | 4.147 | | 0.560 | | 0.000 | | 0.000 | | 0.000 | 0.112 | | 0.224 | | | | 0.560 | | 6.4 | 19.9 |
| Forest | 0.000 | 0.112 | 0.112 | 0.112 | | 0.112 | | 0.000 | | 0.000 | | 0.000 | | 0.000 | 0.000 | | 0.000 | | | | 0.000 | | 0.04 | 0.06 |
| Phosphate P (kg/ha) |  |  |  |  |  | |  | |  | |  | |  | | |  | |  |  |  | |  |  |  |
| Corn/Soy | 0.000 | 0.000 | 0.000 | 0.336 | | 0.448 | | 0.112 | | 0.000 | | 0.000 | | 0.000 | 0.000 | | 0.000 | | | | 0.000 | | 0.07 | 0.15 |
| Corn | 0.112 | 0.112 | 0.000 | 0.785 | | 0.897 | | 0.112 | | 0.000 | | 0.000 | | 0.000 | 0.000 | | 0.112 | | | | 0.112 | | 0.19 | 0.31 |
| Forest | 0.000 | 0.000 | 0.000 | 0.000 | | 0.000 | | 0.000 | | 0.000 | | 0.000 | | 0.000 | 0.000 | | 0.000 | | | | 0.000 | | 0.00 | 0.00 |
| Organic P (kg/ha) |  |  |  |  | |  | |  | |  | |  | |  |  | |  | | | |  | |  |  |
| Corn/Soy | 0.000 | 0.000 | 0.000 | 1.009 | | 2.018 | | 0.897 | | 0.112 | | 0.112 | | 0.673 | 0.000 | | 0.000 | | | | 0.000 | | 0.40 | 0.63 |
| Corn | 0.000 | 0.000 | 0.000 | 2.130 | | 5.044 | | 0.560 | | 0.000 | | 0.112 | | 0.224 | 0.000 | | 0.000 | | | | 0.000 | | 0.67 | 1.51 |
| Forest | 0.000 | 0.000 | 0.000 | 0.000 | | 0.000 | | 0.000 | | 0.000 | | 0.000 | | 0.000 | 0.000 | | 0.000 | | | | 0.000 | | 0.00 | 0.00 |
| Sediment (kg/ha) |  |  |  |  |  | |  | |  | |  | |  | | |  | |  |  |  | |  |  |  |
| Corn/Soy | 0.0 | 0.0 | 0.0 | 1,121 | | 2,466 | | 1,345 | | 0.0 | | 224 | | 1,345 | 0.0 | | 0.0 | | | | 0.0 | | 541.7 | 824.8 |
| Corn | 0.0 | 0.0 | 0.0 | 2,242 | | 4,708 | | 448 | | 0.0 | | 0.0 | | 224 | 0.0 | | 0.0 | | | | 0.0 | | 635.1 | 1433.3 |
| Forest | 0.0 | 0.0 | 0.0 | 0.0 | | 0.0 | | 0.0 | | 0.0 | | 0.0 | | 0.0 | 0.0 | | 0.0 | | | | 0.0 | | 0.00 | 0.00 |

# Farming Operations

Table C. Corn Farming Operations

| Operation | Month | Day | Type | Amount | Units | N (%) | P  (%) |
| --- | --- | --- | --- | --- | --- | --- | --- |
| Tillage | April | 5 | Chisel Plow |  |  |  |  |
| Fertilizer | April | 15 | Fertilizer | 201.6 | kg/ha | 100 |  |
| Fertilizer | April | 15 | Fertilizer | 67.2 | kg/ha |  | 100 |
| Tillage | May | 4 | Field Cultivator |  |  |  |  |
| Planting | May | 5 | Regular Planter | 0.086 | seeds/m^2^ |  |  |
| Harvest | October | 10 |  |  |  |  |  |
| End crop season | October | 11 |  |  |  |  |  |
| Tillage | October | 18 | Tandem Disk |  |  |  |  |

Table D. Corn/Soybean rotation Farming Operations

| Operation | Year | Month | Day | Type | Amount | Units | N (%) | P  (%) |
| --- | --- | --- | --- | --- | --- | --- | --- | --- |
| Tillage | 1 | April | 5 | Chisel Plow |  |  |  |  |
| Fertilizer | 1 | April | 15 | Fertilizer | 201.6 | kg/ha | 100 |  |
| Fertilizer | 1 | April | 15 | Fertilizer | 67.2 | kg/ha |  | 100 |
| Tillage | 1 | May | 4 | Field Cultivator |  |  |  |  |
| Plant corn | 1 | May | 5 | Regular Planter | 10.0 | seeds/m^2^ |  |  |
| Harvest | 1 | October | 10 |  |  |  |  |  |
| End crop season | 1 | October | 11 |  |  |  |  |  |
| Tillage | 1 | October | 18 | Tandem Disk |  |  |  |  |
| Tillage | 2 | April | 5 | Chisel Plow |  |  |  |  |
| Fertilizer | 2 | May | 4 | Field Cultivator |  |  |  |  |
| Tillage | 2 | May | 14 | Fertilizer | 44.8 | kg/ha |  | 100 |
| Plant soybean | 2 | May | 15 | Regular Planter | 76 | seeds/m^2^ |  |  |
| Harvest | 2 | October | 15 |  |  |  |  |  |
| End crop season | 2 | October | 16 |  |  |  |  |  |
| Tillage |  | October | 18 | Tandem Disk |  |  |  |  |

Table E. Forest Farming Operations

| Operation | Month | Day | Type | Amount | Units | N (%) | P  (%) |
| --- | --- | --- | --- | --- | --- | --- | --- |
| Planting | April | 5 | Transplanter | 0.11 | trees/m^2^ | 0 | 0 |

Table F. Data from the analysis of 1,653 soil types and weather conditions, used to create Figure 5

|  | TN | TN | TP | TP | Sediment | Sediment |
| --- | --- | --- | --- | --- | --- | --- |
|  | Crop | Forest | Crop | Forest | Crop | Forest |
|  | (kg/ha) | (kg/ha) | (kg/ha) | (kg/ha) | (kg/ha) | (kg/ha) |
|  |  |  |  |  |  |  |
| Min | 30.7 | 0.7 | 1.0 | 0.000 | 45 | 0.000 |
| Q1 | 41.1 | 2.6 | 1.6 | 0.002 | 179 | 0.000 |
| Median | 64.6 | 6.3 | 2.2 | 0.008 | 426 | 0.000 |
| Q3 | 97.3 | 25.7 | 4.7 | 0.118 | 1702 | 0.011 |
| Max | 179.1 | 69.4 | 10.1 | 0.300 | 5835 | 0.123 |

# References

1. Li C, Trettin C, Sun G, McNulty S, Butterbach-Bahl K. Modeling carbon and nitrogen biogeochemistry in forest ecosystems. 2005. p. 3rd International Nitrogen Conference: 893-898. Available: http://www.treesearch.fs.fed.us/pubs/25254

2. Aber JD, Reich PB, Goulden ML. Extrapolating leaf CO2 exchange to the canopy: a generalized model of forest photosynthesis compared with measurements by eddy correlation. Oecologia. 1996;106: 257–265. doi:10.1007/BF00328606

3. Li C, Frolking S, Frolking TA. A model of nitrous oxide evolution from soil driven by rainfall events: 1. Model structure and sensitivity. J Geophys Res Atmos. 1992;97: 9759–9776. doi:10.1029/92JD00509

4. Li C, Frolking S, Harriss R. Modeling carbon biogeochemistry in agricultural soils. Global Biogeochem Cycles. 1994;8: 237–254. doi:10.1029/94GB00767

5. Thornton PE, Running SW, Hunt ER. Biome-BGC: Terrestrial Ecosystem Process Model, Version 4.1.1, doi:10.3334/ORNLDAAC/805 [Internet]. 2005. Available: https://daac.ornl.gov/cgi-bin/dsviewer.pl?ds_id=805

6. Running SW, Coughlan JC. A general model of forest ecosystem processes for regional applications I. Hydrologic balance, canopy gas exchange and primary production processes. Ecol Modell. 1988;42: 125–154. doi:10.1016/0304-3800(88)90112-3

7. Kimmins JP, Mailly D, Seely B. Modelling forest ecosystem net primary production: the hybrid simulation approach used in forecast. Ecol Modell. 1999;122: 195–224. doi:10.1016/S0304-3800(99)00138-6

8. Bachelet D, Lenihan J, Daly C, Neilson R, Ojima D, Parton W. MC1: A dynamic vegetation model for estimating the distribution of vegetation and the associated ecosystem fluxes of carbon, nutrients and water. Gen. Tech. Rep. PNW-GTR-508. Portland, OR; 2001.

9. Neilson R. A Model for Predicting Continental-Scale Vegetation Distribution and Water Balance. Ecol Appl. 1995;5: 362–385. Available: http://www.jstor.org/stable/1942028?seq=1#page_scan_tab_contents

10. Parton W, Schimel D, Ojima D, Cole C. A general model for soil organic matter dynamics: sensitivity to litter chemistry, texture and management. Pages 147-167 in R.B. Bryant and R.W. Arnold, editors. Quantitative modeling of soil forming processes. SSSA Spec. Publ. 39. 1994.

11. Parton WJ, Schimel DS, Cole C V., Ojima DS. Analysis of Factors Controlling Soil Organic Matter Levels in Great Plains Grasslands1. Soil Sci Soc Am J. Soil Science Society of America; 1987;51: 1173. doi:10.2136/sssaj1987.03615995005100050015x

12. Arnold JG, Fohrer N. SWAT2000: current capabilities and research opportunities in applied watershed modelling. Hydrol Process. 2005;19: 563–572. Available: http://dx.doi.org/10.1002/hyp.5611

13. Green CH, van Griensven A. Autocalibration in hydrologic modeling: Using SWAT2005 in small-scale watersheds. Environ Model Softw. Elsevier; 2008;23: 422–434. doi:10.1016/J.ENVSOFT.2007.06.002

14. Watson B, Coops N, Selvalingam S, Ghafouri M. Integration of 3-PG into SWAT to simulate the growth of evergreen forests. SWAT 2005 3rd Int SWAT Conf. USDA-ARS Research Lab; 2005; 142–152. Available: http://dro.deakin.edu.au/view/DU:30014646

15. Y. Sui Y, J. R. Frankenberger JR. Nitrate Loss from Subsurface Drains in an Agricultural Watershed Using SWAT2005. Trans ASABE. American Society of Agricultural and Biological Engineers; 2008;51: 1263–1272. doi:10.13031/2013.25243

16. Gassman PW, Reyes MR, Green CH, Arnold JG. The Soil and Water Assessment Tool: Historical Development, Applications, and Future Research Directions. Trans ASABE. American Society of Agricultural and Biological Engineers; 2007;50: 1211–1250. doi:10.13031/2013.23637

17. Williams J, Sharpley A. EPIC-erosion/productivity impact calculator: 1. Model documentation technical bulletin no 1768. Washington DC; 1989.

18. Williams JR, Arnold J, Srinivasan R. The APEX Model. BRC Report No. 00-06. Temple, TX; 2000.

19. Johnson M-V V, Macdonald JD, Kiniry JR, Arnold J. ALMANAC: A potential tool for simulating agroforestry yields and improving SWAT simulations of agroforestry watersheds. Int Agric Eng J. 2009;18: 51–58. Available: https://www.researchgate.net/publication/228626733_ALMANAC_A_potential_tool_for_simulating_agroforestry_yields_and_improving_SWAT_simulations_of_agroforestry_watersheds

20. MacDonald JD, Kiniry JR, Putz G, Prepas EE. A multi-species, process based vegetation simulation module to simulate successional forest regrowth after forest disturbance in daily time step hydrological transport models. J Environ Eng Sci. 2008;7: 127–143. doi:10.1139/S08-008

21. Gassman P, Williams J, Wang X, Saleh A, Edward Osei E, Hauck L, et al. The Agricultural Policy Environmental EXtender (APEX) Model: An Emerging Tool for Landscape and Watershed Environmental Analyses, Technical Report 09-TR 49. Ames, Iowa 50011-1070; 2009.

22. Kumar S, Udawatta RP, Anderson SH, Mudgal A. APEX model simulation of runoff and sediment losses for grazed pasture watersheds with agroforestry buffers. Agrofor Syst. 2010;83: 51–62. doi:10.1007/s10457-010-9350-7

23. P. Tuppad P, C. Santhi C, X. Wang X, J. R. Williams JR, R. Srinivasan R, P. H. Gowda PH. Simulation of Conservation Practices Using the APEX Model. Appl Eng Agric. American Society of Agricultural and Biological Engineers; 2010;26: 779–794. doi:10.13031/2013.34947

24. Saleh, A., J.R. Williams JR, J.C. Wood JC, L. M. Hauck LM, W.H. Blackburn WH. Application of APEX for Forestry. Total Maximum Daily Load (TMDL): Environmental Regulations, Proceedings of 2002 Conference. St. Joseph, MI: American Society of Agricultural and Biological Engineers; 2002. p. 595. doi:10.13031/2013.7614

25. Azevedo JC, Wu X Ben, Messina MG, Fisher RF. Assessment of Sustainability in Intensively Managed Forested Landscapes: A Case Study in Eastern Texas. For Sci. Oxford University Press; 2005;51: 321–333. doi:10.1093/forestscience/51.4.321

26. Azevedo JC, Williams JR, Messina MG, Fisher RF. Impacts of the Sustainable Forestry Initiative Landscape Level Measures on Hydrological Processes. Water Resour Manag. Kluwer Academic Publishers; 2005;19: 95–110. doi:10.1007/s11269-005-1503-5
